# Electrolyte imbalance in infectious disease patients at King Abdulaziz Hospital, Jeddah

**DOI:** 10.1016/j.jtumed.2021.09.010

**Published:** 2021-11-20

**Authors:** Fatma I. Albeladi, Iman M. Wahby Salem, Albandari A. Albandar, Hamidah A. Almusaylim, Ali S. Albandar

**Affiliations:** a Internal Medicine Department (Nephrology), King Abdulaziz university, Faculty of Medicine, Family and Community Medicine, Rabigh, KSA; b Community and Occupational Medicine, Faculty of Medicine, Al Azhar University, Egypt; c Faculty of Medicine, King Abdulaziz University, Jeddah, KSA; d Oral and Maxillofacial Surgery, Faculty of Medicine, King Abdulaziz University, Jeddah, KSA

**Keywords:** الخلل بتوازن الكهارل, الاستشفاء, الأمراض المعدية, صوديوم, بوتاسيوم, Electrolyte imbalance, Hospital stay, Infectious diseases, Potassium, Sodium

## Abstract

**Objectives:**

Infectious diseases are the common cause of morbidity and mortality among humans. Electrolyte imbalance occurs frequently in patients with infectious diseases. This study aims to identify electrolyte imbalances in hospitalised patients with infectious diseases.

**Methods:**

Two hundred and eighty-three patients with age mean 36.48 ± 18.86 years, consisting of 127 (53.4%) males, 111 (46.6%) females, enrolled in a retrospective cohort study carried out at the King Abdulaziz University Hospital, Jeddah, KSA from September to December 2020. All hospitalised patients with infectious diseases were included. Demographic data, comorbidity, and diagnosis were collected from patients’ sheets. Serum levels of electrolytes (chloride, potassium, sodium), urea, and creatinine were collected at admission (period 1), during hospital stay (period 2), and at discharge (period 3). Levels were compared during different periods.

**Results:**

Most infectious diseases were viral infections (63.4%), while comorbidity was diabetes mellitus (7.1%). Serum chloride elevated from period 1 to period 3 (P = 0.046). Sodium elevated between period 1 and both period 2 and period 3 (P < 0.001). Urea decreased between period 1 and both period 2 (P = 0.018) and period 3 (P < 0.001). Creatinine decreased between period 1 and both period 2 and period 3 (P < 0.001) and between period 2 and period 3 (P < 0.001). Patients with decreased chloride and sodium levels were mostly in the 1st period, while those with decreased potassium levels were mostly in the period 2.

**Conclusion:**

Prevalence of electrolyte imbalance in hospitalised patients with an infectious disease at the King Abdulaziz University Hospital, Jeddah was high, especially at admission and during the hospital stay.

## الملخص

**أهداف البحث:**

الأمراض المعدية سبب شائع للأمراض والوفيات بين الناس. الخلل بتوازن الكهارل يحدث بكثرة لدى مرضى الأمراض المعدية. هدفت هذه الدراسة إلى معرفة الخلل بتوازن الكهارل لدى المرضى المصابين بأمراض معدية بالمستشفيات.

**طرق البحث:**

فى هذه الدراسةالاستعاديةتم إدراج 283مريضا متوسط أعمارهم 36.48 ± 18.86 سنة؛يتألفوا من 127 (53.4٪) من الذكور و111 (46.6٪) من الإناث. أجريت هذه الدراسه بمستشفى الملك عبد العزيز الجامعي في جدة، المملكة العربية السعودية في الفترة من سبتمبر إلى ديسمبر2020. شملت الدراسة جميع المرضى المقيمين المصابين بأمراض معدية في المستشفيات. تم جمع وتسجيل البيانات الديمغرافية، والأمراض المصاحبة وتشخيص الأمراض المعدية من ملفات المرضى. وتم جمع مستويات المصل من الكهارل (الكلوريد والبوتاسيوم والصوديوم)، واليوريا والكرياتينين التي يتم قياسها في وقت الإدخال للمستشفى (الفترة 1)، وأثناء الاستشفاء في المستشفى بعد التشخيص (الفترة 2) وعند الخروج من المستشفى (الفترة 3). وتم مقارنة المستويات خلال الفترات المختلفة.

**النتائج:**

كانت أغلب الأمراض المعدية عبارة عن التهابات فيروسية (63.4%)، في حين كانت الأمراض المصاحبة مرض البول السكري (7.1%). ووجد ارتفاع الكلوريد فى المصل بشكل ملحوظ من الفترة 1 إلى الفترة 3، وارتفع الصوديوم بشكل ملحوظ بين الفترة 1 والفترة 2 والفترة 3، بينما انخفضت اليوريا بشكل ملحوظ بين الفترة 1 وكل من الفترة 2 والفترة 3. وانخفض الكرياتينين بشكل ملحوظ بين الفترة 1 وكل من الفترة 2 والفترة 3، وكذلك بين الفترة 2 والفترة 3. كان عدد المرضى الذين يعانون من انخفاض مستويات الكلوريد والصوديوم في الغالب خلال الفترة الأولى بينما أولئك الذين كانت لديهم مستويات منخفضة من البوتاسيوم كانوا في الغالب في الفترة الثانية.

**الاستنتاجات:**

كان انتشار اختلال توازن الكهارل في مرضى الأمراض المعدية بالمستشفيات في مستشفى الملك عبد العزيز الجامعي بجدة مرتفعا خاصة عند الدخول للمستشفى وأثناء الاستشفاء بالمستشفى. ولذلك يلزم إجراء مزيد من الدراسات لمعرفة العوامل المساهمة المرتبطة باختلال توازن الكهارل أثناء الاستشفاء بالمستشفى.

## Introduction

Infectious diseases have been the major cause of both morbidity and mortality throughout human history. Out of these infectious diseases that lead to death globally, human immunodeficiency virus **(**HIV)/acquired immunodeficiency syndrome (AIDS), acute lower respiratory tract infections, pulmonary tuberculosis, and malaria predominate. In spite of earlier detection to the contrary, infectious diseases remain a common feature of international public health problems for the 21st century due to related complications.^1^

Electrolyte imbalance occurs frequently in patients with infectious diseases; however, there were insufficient studies dealing with this issue. Potassium (K^+^) is the main intracellular cation that has an important role in preserving cell functions. Main potassium function relies on action potential, mostly in excitable tissues such as nerves and muscles.^2^ Potassium normal serum levels are maintained primarily by the kidneys.^2^^,^^3^ Hypokalaemia, which is represented as a serum potassium level <3.5 mmol/L, is a common and serious electrolyte disturbance.^4^ Hypokalaemia incidence in hospitalised cases is 20%, while the incidence of severe hypokalaemia cases (<3.0 mmol/L), is approximately 5%.^2^^,^^5^ Imbalances in K^+^ levels lead to disruption of heart electrical conduction, dysrhythmias, and might lead to sudden death.^2^ The electrolyte responsible for the normal distribution of water and osmotic pressure in body fluids is sodium (Na^+^), which is an extracellular fluid cation. Changes in normal serum sodium levels are associated with various disorders.^6^ An increase in sodium levels occurs due to inadequate water intake, diarrhoea, or dehydration and this leads to disturbance in brain function, such as convulsion and abnormalities in conscious level, but its decrease results from inadequate water excretion and leads to brain oedema.^7^ A literature review reported hyponatremia in Ebola-infected patients, COVID-19 patients, and influenza-infected patients.^8^ Chloride (Cl^−^) ions are the most common anions present in extracellular fluid.^9^ Chloride ions play important roles in maintaining several physiologic actions like acid-base balance, hydrochloric acid formation in the stomach, and cellular electrolyte homeostasis.^10^ Although chloride is one of the main electrolytes reported in the basic chemistry panel, physicians often overlook the importance of dyschloremia in clinical practice.^11^ Hypocalcaemia, hyponatraemia, hypomagnesaemia, and hyperkalaemia usually occur in malaria.^12^

There are few published studies showing the relationship between electrolyte imbalances in infectious disease patients. Thus, the aim of this retrospective cohort study was to investigate electrolyte imbalances in infectious disease patients at the King Abdulaziz University hospital (KAUH), Jeddah.

## Material and Methods

A retrospective cohort study from September to December 2020 included 317 hospitalised patients diagnosed with infectious diseases at the Internal Medical department at KAUH, Jeddah, KSA. Two hundred and thirty-eight patients were included with a mean age of 36.48 years old. The patients included 127 (53.4%) males and 111 (46.6%) females. The patients included in this study were all hospitalised patients with infectious diseases. Excluded from the study were patients on medications that interfere with potassium levels, such as insulin, angiotensin II receptor blockers (ARBs), angiotensin-converting enzyme inhibitors (ACEI) and diuretic drugs and patients who had hypokalaemia, shock, congestive heart failure, renal insufficiency, and diarrhoea at admission. Also, we excluded from the study patients with psychiatric or neurological diseases and patients on psychiatric or neurological medications that affected electrolytes levels. After applying the exclusion criteria, only 238 patients were included in this study.

The following information was execrated from medical records of each case: demographic data, such as MRN, age, gender, date of birth (DOB) and nationality, comorbidity before infections [diabetes mellitus (DM), hepatobiliary disorder, urinary tract disorders, chronic obstructive pulmonary disease (COPD), hypertension, asthma, thyroid diseases, and sickle cell anaemia (SCA)]. The diagnosis of infectious diseases was also recorded [Typhoid fever, Dengue fever, viral infection, tuberculosis, non-diabetic foot ulcer, cholecystitis, urinary tract infection (UTI), and systemic bacterial infections]. Also, clinical data were extracted from the reports, including vital signs [heart rate, body temperature, respiratory rate, and blood pressure], electrolytes levels (potassium, chloride, and sodium) as well as urea and creatinine during three periods, period 1 (at admission), period 2 (during hospital stay) and period 3 (at discharge).

Period 2 included electrolytes readings taken by patients during their hospital stays, which were repeated numerous times. For example, if a patient made electrolyte reading at least once a day, the patient had a total of seven electrolytes readings per week; a mean of seven readings was calculated at the weekend and taken as a level of that week. This problem was handled by randomising all data by taking an average reading during the hospital stay, collecting all of the readings, and then dividing the numbers of readings to obtain the mean.

### Statistical analysis

The data were collected using Google forms service and coded and processed using Microsoft Excel. The Statistical Package for the Social Science (SPSS) Software version 23 (IBM SPSS, IBM Corp., Armonk, N.Y., USA). Descriptive statistics, including number, percentages (%), mean, ±standard deviation (SD), were used to describe items and study variables. Repeated one-way ANOVA using post hoc (Bonferroni test) was conducted to test differences of change across periods. *P* values <0.05 were considered statistically significant.

## Results

As shown in Table 1, among the participants, 164 (68.9%) were Saudi and 74 (31.1%) were non-Saudi. Of patients, 17 (7.1%) had diabetes mellitus and 10 (4.2%) hypertension, while only five (2.1%) had thyroid diseases, two (0.8%) had sickle cell anaemia (SCA), and only one patient (0.4%) had bronchial asthma.

**Table 1:** Demographic data and descriptive of the diseases of all patients (N = 238).

Factors	Value
**Age** (years)	36.48 ± 18.86
**Gender**	
Male	127 (53.4%)
Female	111 (46.6%)
**Nationality**	
Saudi	164 (68.9%)
Non Saudi	74 (31.1%)
**Comorbidity**	
No disease	211 (88.7%)
All comorbidities	27 (11.3%)
*Diabetes mellitus*	17 (7.1%)
*Hypertension*	10 (4.2%)
*Thyroid diseases*	5 (2.1%)
*Sickle cell anaemia*	2 (0.8%)
*Asthma*	1 (0.4%)

Data were expressed as mean ± standard deviation or number (%) as appropriate.

The infectious diseases were mostly viral infections (n = 151, 63.4%) followed by tuberculosis (n = 60, 25.21%), cholecystitis (n = 19, 8.0%), and systemic bacterial infections (n = 8, 3.3%) (Table 2).

**Table 2:** Underlying infectious diseases (N = 238).

Diagnosis	Number (%)
Viral infections	151 (63.4%)
Tuberculosis	60 (25.2%)
Cholecystitis	19 (8.0%)
Systemic bacterial infections	8 (3.3%)

**Figure 1:**
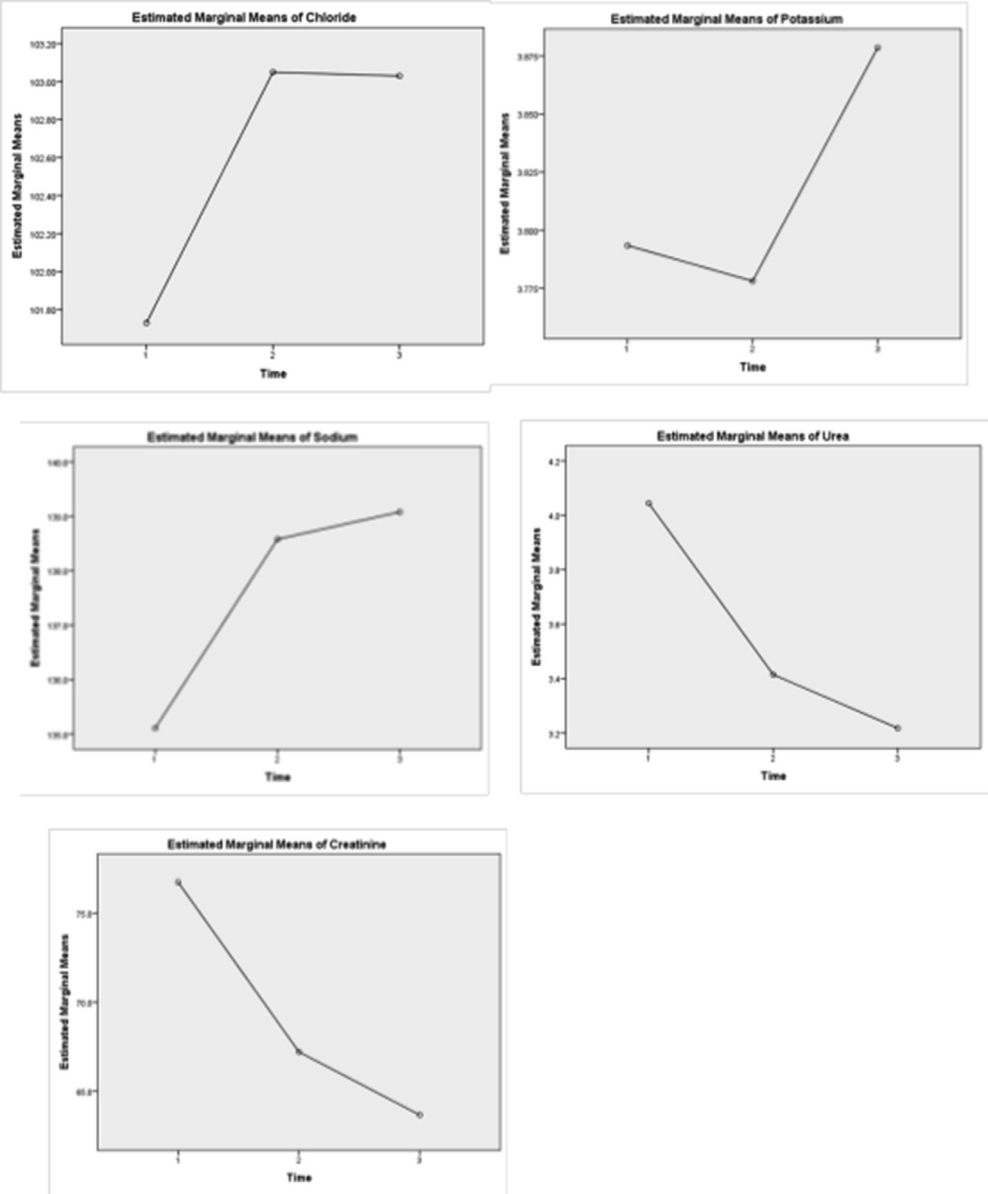
Repeated measure ANOVA test for measured electrolytes, urea and creatinine at different period of time (N = 238).

A repeated measure ANOVA was conducted to investigate the effect of diagnosis over three periods, period 1 (at admission), period 2 (during hospital stay), and period 3 (at discharge) of five elements, present in Table 3, Table 4 and Figure 1. The Greenhouse-Geisser correction determined that the mean chloride value change was statistically significant between periods (df: 2, 87.632, F = 4.521, P = 0.015). Post hoc tests utilizing the Bonferroni correction showed that diagnosis had a slight increase in Cl^−^ concentration from period 1 to period 2 (101.73 ± 6.42 vs, 103.05 ± 5.07 mmol/L respectively), which was not statistically significant (P = 0.058). However, at period 3, it increased to 103.03 ± 4.46 mmol/L, which was statistically significantly different from period 1 concentration (P = 0.046). Therefore, it can be concluded that a long-term diagnosis between period 1 and period 3 elicits a statistically significant increase in Cl^−^ concentrations. The Greenhouse-Geisser correction determined that mean potassium value did not differ statistically significantly between periods (df: 1.87, 0.429, F = 2.507, P = 0.087). Post hoc tests revealed that diagnosis had a slight decrease in K^+^ concentration from period 1 to period 2 (3.793 ± 0.438 versus 3.778 ± 0.528 mmol/L respectively). However, in period 3, it increased to 3.879 ± 0.366 mmol/L, which was insignificantly different from period 1 and period 2 concentrations; therefore, it can be concluded that a long-term diagnosis between period 1 and period 3 elicits an insignificant increase in K^+^ concentration. The Greenhouse-Geisser correction determined that mean sodium value change was statistically significant between periods (df: 1.982, 651.280, F = 76.430, P < 0.0001). Post hoc tests utilizing Bonferroni correction revealed that diagnosis had increased in Na^+^ concentration from period 1 to period 2 and period 3 (135.109 ± 3.881 versus 138.580 ± 4.090 versus, 139.080 ± 3.498 mmol/L respectively), which was statistically significant between period 1 compared to period 2 (P < 0.0001) and period 1 versus period 3 (P < 0.0001). It can be concluded that a long-term diagnosis between times (1, 2, and 3) elicits a statistically significant increase in Na^+^ concentration, especially till diagnosis. The Greenhouse-Geisser correction determined that the mean urea value change was statistically significantly different between periods (df: 1.736–29.396, F = 10.121, P < 0.0001). The diagnosis had a significant reduction in urea concentration from period 1 to period 2 (4.044 ± 2.601 versus 3.415 ± 2.895 mmol/L, P = 0.018). However, in period 3, it slightly decreased to 3.218 ± 2.348 mmol/L, which was statistically different from period 1 concentrations (P < 0.0001) but insignificantly differ from period 2 (P = 0.761). Long-term diagnosis between period 1 and period 2 elicits a statistically significant decrease in urea concentration, but not after period 2 of diagnosis. The Greenhouse-Geisser correction determined that the mean creatinine value change was statistically significant in different periods (df: 1.710–7334.315, F = 31.138, P < 0.0001). The diagnosis had a reduction in creatinine concentration from period 1 to period 2 (76.738 ± 38.152 versus 67.20 ± 35.276 umol/L respectively), which was statistically significant (P < 0.0001). However, in period 3, it slightly decreased to 63.655 ± 33.424 umol/L, which was statistically different from period 1 (P < 0.0001) and period 2 (P = 0.027) concentrations. Long-term diagnosis between periods 1, 2, and 3 elicits a statistically significant decrease in creatinine concentration.

**Table 3:** Repeated measure ANOVA test for measured electrolytes, urea and creatinine at different period of time (N = 238).

Factor	Period	Mean	SD	Greenhouse- Geisser			Sig.
				df (mean Square)		F	
Chloride	Period 1	101.73	6.42	2	87.632	4.521∗	**0.015**
	Period 2	103.05	5.07				
	Period 3	103.03	4.46				
Potassium	Period 1	3.793	0.438	1.87	0.429	2.507	0.087
	Period 2	3.778	0.528				
	Period 3	3.879	0.366				
Sodium	Period 1	135.109	3.881	1.982	651.280	76.430∗∗∗	**0.0001**
	Period 2	138.580	4.090				
	Period 3	139.080	3.498				
Urea	Period 1	4.044	2.601	1.736	29.396	10.121∗∗∗	**0.0001**
	Period 2	3.415	2.895				
	Period 3	3.218	2.348				
Creatinine	Period 1	76.738	38.152	1.710	7334.315	31.138∗∗∗	**0.0001**
	Period 2	67.200	35.276				
	Period 3	63.655	33.424				

∗: P < 0.05, ∗∗∗: P < 0.001.

**Table 4:** Post hocs multiple comparisons) (Bonferroni test) for measured electrolytes, urea and creatinine at different period of time (N = 238).

Dependent Variable			Mean Difference	Std. Error	Sig.	95% Confidence Interval	
						Lower Bound	Upper Bound
Chloride	Period 1	Period 2	−1.319	0.558	0.058	−2.670	0.032
		Period 3	−1.299∗	0.529	**0.046**	−2.582	−0.016
	Period 2	Period 3	0.20	0.409	1.000	−0.971	1.011
Potassium	Period 1	Period 2	0.015	0.054	1.000	−0.116	0.147
		Period 3	−0.085	0.044	0.161	−0.191	0.021
	Period 2	Period 3	−0.101	0.047	0.098	−0.213	0.012
Sodium	Period 1	Period 2	−3.471∗∗∗	0.366	**0.0001**	−4.358	−2.584
		Period 3	−3.971∗∗∗	0.340	**0.0001**	−4.794	−3.148
	Period 2	Period 3	−0.500	0.344	0.444	−1.333	0.333
Urea	Period 1	Period 2	0.629∗	0.226	**0.018**	0.081	1.178
		Period 3	0.826∗∗∗	0.173	**0.0001**	0.408	1.245
	Period 2	Period 3	0.197	0.172	0.761	−0.219	0.613
Creatinine	Period 1	Period 2	9.538∗∗∗	1.964	**0.0001**	4.778	14.298
		Period 3	13.083∗∗∗	1.780	**0.0001**	8.768	17.398
	Period 2	Period 3	−3.545∗	1.341	**0.027**	−6.795	−0.295

∗: P < 0.05, ∗∗∗: P < 0.001.

Patients with decreased chloride serum levels (<98 mmol/L) in period 1 were 32 (13.4%), 17 (7.1%) in period 2, and 16 (6.7%) in period 3. Patients with increased chloride serum levels (>107 mmol/L) in period 1 were 19 (8.0%), a number rose to 35 (14.7%) in period 2, and then decreased to 18 (7.6%) in period 3. Patients with decreased potassium serum levels (<3.5 mmol/L) in period 1 were 41 (17.2%), a number increased to 46 (19.3%) in period 2, and decreased to 19 (80%) in period 3. Patients with increased potassium serum levels (>5.1 mmol/L) in period 1 were 2 (0.8%), and the number rose to 5 (2.1%) in period 2. Patients with decreased in sodium serum levels (<136 mmol/L) in period 1 were 103 (43.3%), a number decreased to 38 (16.0%) in period 2, and to 22 (9.2%) at period 3. Patients with increased chloride serum levels (>145 mmol/L) in period 2 were 5 (2.1%) and their number decreased to 1 (0.4%) in period 3. Patients with decreased urea serum levels (<2.5 mmol/L) in period 1 were 53 (22.3%), their number increased to 71 (29.8%) in period 2 and 64 (26.9%) in period 3. Patients with increased urea serum levels (>6.4 mmol/L) at period 1 were 20 (8.4%), their decreased to 12 (5.0%) in period 2, and then decreased to 7 (2.9%) in period 3. Patients with decreased creatinine serum levels (<53 umol/L) at period 1 were 61 (25.6%), their number decreased to 59 (24.8%) in period 2 and 58 (24.4%) in period 3. Patients with increased creatinine serum levels (>115 umol/L) at period 1 were 13 (5.5%) and their number decreased to 5 (2.1%) in period 2 (Table 5).

**Table 5:** Serum electrolytes, urea and creatinine levels during different **period of time** (N = 238).

Electrolytes	Period 1	Period 2	Period 3
**Chloride serum levels** (normal range 98–107 mmol/L)			
Low (<98 mmol/L)	32 (13.4%)	17 (7.1%)	16 (6.7%)
High (>107 mmol/L)	19 (8.0%)	35 (14.7%)	18 (7.6%)
**Potassium serum levels** (normal range 3.5–5.1 mmol/L)			
Low (<3.5 mmol/L)	41 (17.2%)	46 (19.3%)	19 (8.0%)
High (>5.1 mmol/L)	2 (0.8%)	5 (2.1%)	–
**Sodium serum levels** (normal range 136–145 mmol/L)			
Low (<136 mmol/L)	103 (43.3%)	38 (16.0%)	22 (9.2%)
High (>145 mmol/L)	–	5 (2.1%)	1 (0.4%)
**Urea serum levels** (normal range 2.5–6.4 mmol/L)			
Low (<2.5 mmol/L)	53 (22.3%)	71 (29.8%)	64 (26.9%)
High (>6.4 mmol/L)	20 (8.4%)	12 (5.0%)	7 (2.9%)
**Creatinine serum levels** (normal range 53–115 umol/L)			
Low (<53 umol/L)	61 (25.6%)	59 (24.8%)	58 (24.4%)
High (>115 umol/L)	13 (5.5%)	5 (2.1%)	–

Data were expressed as number (%).

## Discussion

This study investigates the electrolytes imbalance in patients with infectious diseases in three consecutive periods, which were defined as period 1 (electrolyte levels at admission), period 2 (electrolyte levels during hospital stay), and period 3 (electrolyte levels at discharge). Infectious diseases in this study include viral infections (63.4%), tuberculosis (25.2%), cholecystitis (8.0%), and systemic bacterial infections (3.3%).

The data from this study showed that at admission 17.2% of patients had hypokalaemia that increased to 19.3% during the hospital stay and decreased to 8.0% at discharge. Meanwhile, at admission, 0.8% had hyperkalaemia that increased to 2.1% during the hospital stay. Moreover, there were insignificant differences in the potassium serum levels at admission, during the hospital stay, and at discharge. Several factors affect K^+^ balance in the body, such as dietary intake of potassium, losses of potassium in urine or through the gastrointestinal tract, and trans-cellular potassium shift.^13^ Inadequate dietary intake was the most common cause of hypokalaemia in infectious diseases patients.^14^ The studies in Jakarta at Cipto Mangunkusumo Hospital determined that hypokalaemia prevalence in hospitalised patients having infectious diseases was 23% at admission and 52.4% on discharge.^15^ Hypokalaemia is managed with oral or intravenous potassium administration and/or potassium-sparing diuretics.^16^ Several studies reported a relationship between low potassium levels and tuberculosis disease. Kardalas et al. stated that tuberculosis patients receiving capreomycin drug experienced electrolytes imbalance.^2^ In 2006, a study conducted by Akinwale et al.^17^ reported that approximately 37% of typhoid fever patients needed ICU care and experienced electrolyte imbalance during their hospital stay.^5^ A recently published paper by Chen et al.^18^ stated that the COVID-19 virus has a strong association with a decrease in a potassium level due to impaired renin-angiotensin system activity^19^ that is somehow consistent with the results of this study that stated that patients with viral infection developed hypokalaemia.

A potential risk of hypokalaemia medications is the occurrence of subsequent hyperkalaemia, which leads to dangerous cardiac irregularities. Potassium alone administration does not lead to hyperkalaemia.^20^ The K^+^ shift into extracellular compartment due to breakdown of tissues during infectious diseases lead to hyperkalaemia.^14^ Two possible sequences are a decrease in renal potassium excretion and a shift hypokalaemia, which often leads to rebound hyperkalaemia.^21^ Drugs suppressing the renin-angiotensin system or blocking aldosterone receptors are linked with hyperkalaemia.^22^ but they were not present in this study as cases on such medications were excluded.

The data from this study showed that there was a significant decrease in serum sodium levels at admission, then the levels increased significantly during the hospital stay period and at discharge. Also, at admission, 43.3% of patients had hyponatremia that decreased to 16.0% during the hospital stay and decreased to 9.2% at discharge. Meanwhile, during the hospital stay, 2.1% of patients developed hypernatremia that decreased to 0.4% at discharge. Hyponatremia is a common electrolyte disorder that develops frequently in 15–40% of hospitalised patients.^23^ Hyponatremia during infection may be transient and may be overlooked by clinicians as it did not lead to specific manifestations. Nonetheless, hyponatremia in infections indicated underlying disease severity and is linked to delayed hospitalization and significant morbidity.^23^ In the majority of cases, causes of hyponatremia are multifactorial. However, increased levels of antidiuretic hormone (ADH), which can be either appropriate, in cases of volume depletion, or inappropriate in cases of inappropriate ADH secretion (SIADH), play the most important role in hyponatremia occurrence in infectious diseases. Under these circumstances of increased ADH release, intake of hypotonic fluids is avoided in order to decrease the incidence of infections inducing hyponatremia.^24^ Hypernatremia that occurred after patients were hyponatremic may be due to improvement of patient's general condition after adequate infection treatment.

The results of this research showed that there was a significant decrease in serum chloride values at admission, then levels increased significantly during the hospital stay period and at discharge. Also, at admission, 13.4% of patients had hypochloremia that decreased to 7.1% during the hospital stay and decreased to 6.7% at discharge. Meanwhile, at admission, 8.0% had hyperchloremia that increased to 14.7% during the hospital stay and decreased to 7.6% at discharge. Hospital-induced dyschloremia is one of the common electrolyte abnormalities, ranging from 30% to 40% of hospitalisations.^25^, ^26^, ^27^ Formal studies revealed that hyperchloremia leads to renal vasoconstriction via tubuloglomerular feedback and elevation of thromboxane formation.^28^ Dyschloremia is linked with bad patient prognosis, including acute kidney injury and prolonged hospital stay.^25^^,^^29^, ^30^, ^31^ While, hypochloremia, in most cases, is community-acquired, hyperchloremia is usually a hospital-induced problem.^32^ Hospital-acquired changes in chloride homeostasis may be due to either a side effect from diseases complications or therapy given by physicians. Administration of chloride-rich crystalloids is one of the most common reasons of iatrogenic hospital-acquired hyperchloremia. Many animal and human studies revealed that an infusion of 0.9% saline associated with hyperchloremia.^1^^,^^30^ Other hospital-acquired etiologies for dyschloremia include hyperchloremia made by hospital diarrhoea or diabetes insipidus and hypochloremia linked to diuretics intake, congestive heart failure, vomiting, and nasogastric tube drainage.^9^

The results of this research also showed that there were significant increases in serum urea and creatinine levels at admission, then levels decreased significantly during the hospital stay period and at discharge. Also, at admission, 8.4% and 5.5% of patients had an increase in urea and creatinine serum levels that decreased to 5.0% and 2.1% during the hospital stay and decreased to 2.9% and 0.0% at discharge. Renal affection in infectious diseases takes place due to different mechanisms as a direct microbial invasion of the kidney may take place due to septicaemic extend or ascending infections; kidney damage may be due to systemic formation of endotoxin or other toxins and inflammatory cascade stimulation during septicaemia; ischemic destruction may occur from insufficient perfusion produced by septic shock; kidney may be damaged by immunologic pathways activation or by immune complexes due to infectious process. In many cases, more than one of these mechanisms occurs.^1^

### Limitations

This study had some limitations as it includes only one hospital and that made data cannot be generalised. The sample size is little to subdivide the patients into categories according to underlying infectious diseases.

## Conclusion

Prevalence of electrolyte imbalance in hospitalised patients suffering from infectious diseases at the KAUH, Jeddah was high especially at admission and during the hospital stay. In patients with infectious diseases, there are abnormal serum electrolytes levels, such as sodium, potassium, and chloride levels at admission or during their hospital stay or at discharge as well as disturbed kidney functions. The diagnosis of electrolyte disturbances in patients with infectious diseases is a challenge for the clinician because of its frequency, different causes, and implications for prognosis and treatment. Suspicion should be raised by a complete physical examination in those patients with suggestive symptoms or at risk, and then ensured by serum electrolyte analysis. Further studies are needed to determine contributing factors associated with electrolyte imbalance during infectious diseases and hospital stay is recommended. A large prospective study that includes many hospitals and a large sample size of patients with infectious diseases is recommended to determine which infectious disease affects more electrolytes imbalance.

## Source of funding

This research did not receive any specific grant from funding agencies in the public, commercial, or not-for-profit sectors.

## Conflict of interest

The authors have no conflict of interest to declare.

## Ethical approval

This study was approved by the institutional review board (IRB) of King Abdulaziz University Hospital (Reference No 663-19, on 6th November 2019).

## Authors contributions

FIA conceived and designed the study and conducted the research. HAA provided research materials, collected, and organised data. IMWS revised the research methodology, analysed, and interpreted the data. ASA collected the data and wrote the discussion. AAA wrote the initial and final draft of the article and provided logistic support. All authors have critically reviewed and approved the final draft and are responsible for the content and similarity index of the manuscript.
